# Prioritization of the micro-watersheds through morphometric analysis in the Vasishta Sub Basin of the Vellar River, Tamil Nadu using ASTER Digital Elevation Model (DEM) data

**DOI:** 10.1016/j.dib.2018.08.197

**Published:** 2018-09-05

**Authors:** R. Poongodi, S. Venkateswaran

**Affiliations:** Hydrogeology Lab, Department of Geology, Periyar University, Salem 636011, India

## Abstract

The dataset for this article includes morphological analysis of the level to which groundwater potential of the Vasishta River, Salem and Perambalur districts of Tamil Nadu. The method for the computation of morphometric parameters using data Digital Elevation Model (DEM) of the Vasishta River, is also prepared using SRTM (Shuttle Radar Topographic Mission) 90 m resolution data Morphometric parameter linear, aerial and relief limits, such as a bifurcation ratio (Rb), Drainage density (Dd) Stream Frequency (Fs) Elongation ratio (Re), Length of overland flow (Lg), Relief ratio, ruggedness number (Rn) and Slope (sb) of Vasishta Sub Basin (VSB). The relief ratio indicates that the discharge should be considered high priority given to the following micro-watersheds numbers 9,1,15,11 and 10. This data could be very useful to help with sustainable groundwater planning in any similar basins.

**Specification table**

**Table:** 

**Subject area**	Earth Science
**More specific subject area**	Environmental Science, morphometry
**Type of data**	Table and Figure
**How data was Acquired**	USGS website http://www.usgs.gov/, ArcGIS 9.3 Software
**Data format**	Raw, Digitized
**Experimental factors**	The morphometric parameters of all the sub basin were calculated using ArcGIS 9.3 Software.
**Experimental features**	Determination of morphological factor that combines the Vasishta river.
**Data source location**	Vasista River Salem and Perambalur districts of Tamil Nadu State,
**Data accessibility**	All the data are with this article.

**Value of the data**

• The data could be used to decide the morphological characteristics of the Vasishta river area slope.
• The data could be helpful for concerned authorities and policy makers in water measure
• Management.
• The data could be used in management, has groundwater potential.

## 1 Data

The data includes morphometric analysis of the Vasishta River Salem and Perambalur districts of Tamil Nadu, The Survey of India toposheet (SOI) year 1973 and ASTER DEM data. Derived from mathematical formula, see Table 1 (the characteristic investigated are in Table 2, Table 3).

**Table 1:** Morphometric parameters and their mathematical expressions.

**S.No**	**Parameter**	**Formula**	**Previous Work**
**Linear aspect**			
1.	Area (*A)*	Area of the watershed	[2]
2.	Perimeter (*P*)	The perimeter is the total length of the watershed boundary.	[7]
3.	Length (*L*_b_)	Maximum length of the watershed	[2]
4.	Stream Order (*N*_u_)	Hierarchical rank	[3]
5.	Stream Length(*L*_u_)	Length of the stream	[2]
6.	Stream length ratio (Rl)	Rl = *L*_u_ / *L*_u_−1	[4]
7.	Mean Stream Length Ratio (Lsm)	Lsm= *L*_u_/*N*_u_	[2]
8.	Bifurcation ratio (*R*_b_)	*R*_b_ = *N*_u_/*N* (*u* + 1)	[5]
**Areal aspect**			
9.	Drainage density (*D*_d_)	$\mathrm{Dd}={\sum \mathrm{Lu}/A}^{\;}$	[2]
10.	Stream frequency (*F*_s_)	Fs=∑Nu/A	[2]
11.	Elongation ratio (*R*_e_)	*R*_e_ = 1.128 √*A*/*L*	[5]
12.	Length of overflow (*L*_g_)	*L*_g_ = 1/2/2*d*	[2]
13.	Compactness coefficient (*C*_c_)	*C*_c_+ 0.282*P*/√*A*^0.5^	[6]
**Relief aspect**			
14.	Basin relief (*R*)	*R* = *H* – *h*	[5]
15.	Ruggedness number (*Rn*)	*Rn*=*R*×*D*_d_	[5]

**Table 2:** Morphometric parameter of the Vasishta Sub Basin.

Micro Watershed. No	*A* (km^2^)	*P* km	*L*	*N*	N1	Lb (km)	*R*_b_	*D*_d_ (Km/km^2^)	*F*_s_	*D*_t_	*L*_g_	*R*_f_	*R*_e_	*B*_s_	*R*_c_	*C*_c_
1	84.36	51.39	693.01	428	343	12.08	3.54	8.21	5.07	8.33	4.11	0.58	0.60	1.73	0.40	1.58
2	84.36	46.95	393.02	203	159	12.38	3.31	4.66	2.41	4.32	2.33	0.55	0.60	1.82	0.48	1.44
3	122.78	59.56	623.75	348	256	23.15	2.76	5.08	2.83	5.84	2.54	0.23	0.73	4.36	0.43	1.52
4	144.53	70.75	692.89	380	284	20.99	3.32	4.79	2.63	5.37	2.40	0.33	0.79	3.05	0.36	1.66
5	125.15	58.02	394.09	156	111	21.91	2.63	3.15	1.25	2.69	1.57	0.26	0.74	3.84	0.47	1.46
6	133.59	63.82	618.11	321	241	23.09	2.76	4.63	2.40	5.03	2.31	0.25	0.76	3.99	0.41	1.56
7	136.45	67.41	412.63	189	142	29.04	3.09	3.02	1.39	2.80	1.51	0.16	0.77	6.18	0.38	1.63
8	107.77	61.09	236.04	116	92	25.86	3.38	2.19	1.08	1.90	1.10	0.16	0.68	6.21	0.36	1.66
9	58.7	37.28	493.32	313	248	15.23	3.26	8.40	5.33	8.40	4.20	0.25	0.50	3.95	0.53	1.37
10	88.42	41.72	543.61	316	230	13.06	3.12	6.15	3.57	7.57	3.07	0.52	0.62	1.93	0.64	1.25
11	97.71	48.1	663.29	396	307	15.88	3.35	6.79	4.05	8.23	3.39	0.39	0.65	2.58	0.53	1.37
12	83.03	41.28	498.65	275	211	11.31	3.09	6.01	3.31	6.66	3.00	0.65	0.60	1.54	0.61	1.28
13	74.57	42.72	445.99	248	195	12.8	3.08	5.98	3.33	5.81	2.99	0.46	0.57	2.20	0.51	1.40
14	32.27	32.97	205.38	115	89	11.8	2.09	6.36	3.56	3.49	3.18	0.23	0.37	4.31	0.37	1.64
15	36.26	32.9	309.63	185	144	11.74	2.97	8.54	5.10	5.62	4.27	0.26	0.40	3.80	0.42	1.54
16	40.95	33.77	252.48	139	107	11.12	2.30	6.17	3.39	4.12	3.08	0.33	0.42	3.02	0.45	1.49
17	319.88	198.3	946.03	426	319	65.21	2.59	2.96	1.33	2.15	1.48	0.08	1.18	13.29	0.10	3.13

**Table 3:** Micro Watersheds parameter of the VSB.

Micro watershed No	No of stream orders							Total stream Numbers
	1 st order	2 nd order	3 rd order	4 th order	5th order	6th order	7th order	
1	343	68	12	4	1	0	0	428
2	159	34	7	1	0	2	0	203
3	256	69	15	5	2	1	0	348
4	284	73	17	5	1	0	0	380
5	111	33	8	3	1	0	0	156
6	241	58	16	4	2	0	0	321
7	142	36	6	4	1	0	0	189
8	92	21	2	1	0	0	0	116
9	248	51	10	3	1	0	0	313
10	230	65	16	4	1	0	0	316
11	307	68	16	4	1	0	0	396
12	211	46	13	3	1	1	0	275
13	195	36	12	4	1	0	0	248
14	89	18	4	4	0	0	0	115
15	144	32	6	2	1	1	0	185
16	107	23	6	2	0	0	0	139
17	319	75	19	4	0	0	1	426

The quantitative morphometric analysis was carried out in seventeen micro-watersheds of VSB catchment using GIS technique for determining [1] the linear aspects, such as Stream order, Bifurcation ratio, Stream length and aerial aspects such as Stream order(U), Stream length (Lu), Mean Stream length (Lsm), Stream length ratio (Rl), Bifurcation ratio (Rb),Length of overland flow (Lg) drainage density (Dd), stream frequency (Fs), Compactness coefficient (Cc) form factor (Rf), circulatory ratio (Rc), and elongation ratio (Re), Relief ratio (Rh), Ruggedness Number. The prioritization based on different morphometric factor is time-consuming.

## 2 Experimental design, methods and materials

Manual extraction of drainage network, assigning the stream order from a published Survey of India (SOI) topographic map and from georeferenced satellite data for a large area is a time consuming and tedious exercise. To overcome this problem, automatic extraction techniques were used for evaluating the morphometric factor of a basin. Extraction of River basin/watershed boundary and extraction of drainage/stream the Vasistha River basin using ASTER DEM in conjunction with geocodes standard false colour composite remote sensing satellite data.

A multi criteria assessment was used to assimilate all the thematic layers. Individual themes and their consistent groups allocated a knowledge base weightages given depending on their suitability to grip groundwater and their weightages calculated. The process of visually interpreting digitally enhanced imagery attempts to optimize the complementary abilities of the human mind and the computer.

### 2.1 Study area

The Vasistha River lies between 11° 24′0.347′′ and 11° 53′26.496′′ N latitudes and 78° 13′55.211′′E to 78° 58′ 9.969′′E longitudes. The area was bounded by Toposheet numbers (58 I/5,6,7,9,10,11,12,14 and 15) survey of India. Having scale of 1:50000. Toposheet published the year 1973. The Vasistha River study area lies in the Salem and Perambalur districts of Tamil Nadu comprises the part of Vellar River. The main river Vasishta originates from the southern slope of the Kalrayan hills and flow through kurchi, Belur, Pethanaickenpalayam, Attur, Pattuthurai, Thalaivasal, Aragalur, Sitheri, Villages of Salem and Perambalur districts of Tamil Nadu.

**Fig. 1:**
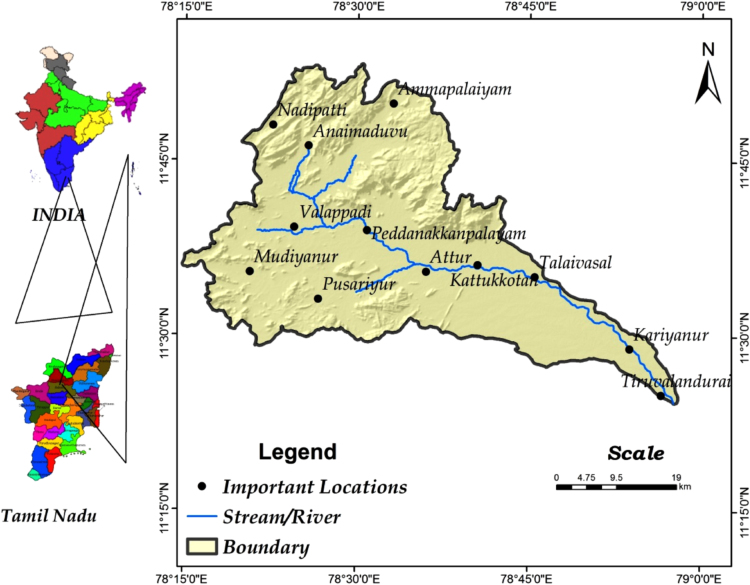
The Base Map of the Vasishta Sub Basin.

It covers an area of 1770.78 km^2^, consisting of the Vasishta Nadi and Sweata Nadi, which drain two parallel valleys running east and west in Attur taluk. Vasishta River runs for a distance of 73 km in Salem, Perambalur and Cuddalore districts and drains into the Bay of Bengal. The climate of the Vasistha River area is mainly sub-tropical climate with moderate humidity and temperature. The VSB is underlaid by the Archaean crystalline rocks surrounded by denudation hills and structural hills.The Base map of the VSB is given in the Fig. 1.

## 3 Compound factor and ranking

**Fig. 2:**
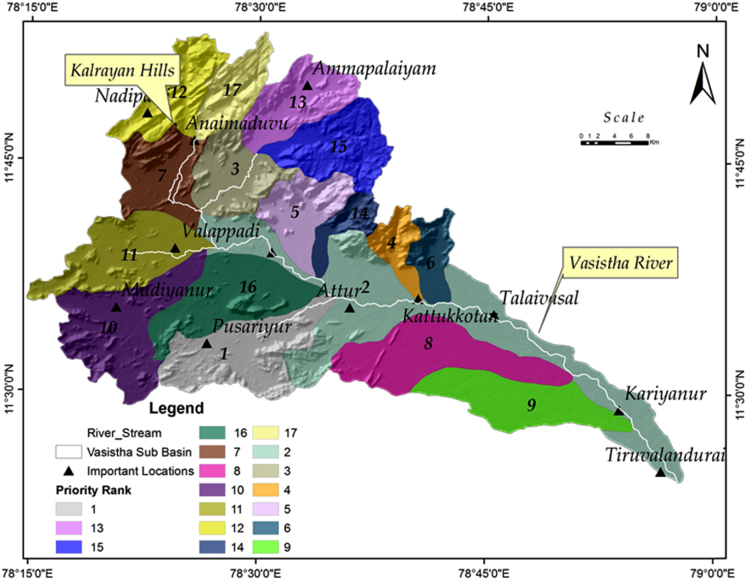
The Priority Map of the Vasishta Sub Basin.

Compound factor is calculated by summing all the ranks of linear, aerial and relief parameter, the shape parameter and then dividing by the number of parameter. From the group of these micro watersheds, highest rank was assigned to the micro watershed having the lowest compound factor and so on. Depending upon the value of compound factor, ranking to each micro watershed assigned in the micro-watershed no. 9 is given as a rank 1 with least compound factor value at 5.2 and it is followed by micro-watersheds no. 1 and 15 as second and third respectively. The values of compound factor and respective rank of all micro- watersheds. The Priority value of the VSB is given in the Fig. 2 and Table 4.

**Table 4:** Prioritization value of the Vasishta Sub Basin.

Micro watershed No	*R*_b_	*D*_d_	*F*_u_	*T*	*L*_o_	*R*_f_	*B*_s_	*R*_e_	*R*_c_	*C*_c_	Compound factor	Prioritization Rank
1	1	3	3	2	3	16	2	7	6	12	5.5	2
2	5	12	12	11	12	15	3	8	12	6	9.6	10
3	12	10	10	6	10	4	14	13	9	9	9.7	11
4	4	11	11	9	11	10	8	17	2	15	9.8	12
5	14	14	16	15	14	8	10	14	11	7	12.3	17
6	13	13	13	10	13	6	12	15	7	11	11.3	13
7	8	15	14	14	15	2	15	16	5	13	11.7	15
8	2	17	17	17	17	3	16	12	3	16	12	16
9	6	2	1	1	2	7	11	5	14	3	5.2	1
10	7	7	5	4	7	14	4	10	17	1	7.6	5
11	3	4	4	3	4	12	6	11	15	4	6.6	4
12	9	8	9	5	8	17	1	9	16	2	8.4	6
13	10	9	8	7	9	13	5	6	13	5	8.5	8
14	17	5	6	13	5	5	13	2	4	14	8.4	7
15	11	1	2	8	1	9	9	3	8	10	6.2	3
16	16	6	7	12	6	11	7	4	10	8	8.7	9
17	15	16	15	16	16	1	17	1	1	17	11.5	14
